# Cancer diagnosis after emergency presentations in people with mental health and substance use conditions: a national cohort study

**DOI:** 10.1186/s12885-024-12292-9

**Published:** 2024-04-30

**Authors:** Ruth Cunningham, James Stanley, Fiona Imlach, Tracy Haitana, Helen Lockett, Susanna Every-Palmer, Mau Te Rangimarie Clark, Cameron Lacey, Kendra Telfer, Debbie Peterson

**Affiliations:** University of Otago, Dunedin, New Zealand

**Keywords:** Cancer, Mental health and substance use disorders, Emergency presentation, Health disparities, Lung neoplasms, Colorectal neoplasms, Breast neoplasms, Prostate neoplasms, Health care access, Diagnostic overshadowing

## Abstract

**Background:**

Cancer survival and mortality outcomes for people with mental health and substance use conditions (MHSUC) are worse than for people without MHSUC, which may be partly explained by poorer access to timely and appropriate healthcare, from screening and diagnosis through to treatment and follow-up. Access and quality of healthcare can be evaluated by comparing the proportion of people who receive a cancer diagnosis following an acute or emergency hospital admission (emergency presentation) across different population groups: those diagnosed with cancer following an emergency presentation have lower survival.

**Methods:**

National mental health service use datasets (2002–2018) were linked to national cancer registry and hospitalisation data (2006–2018), to create a study population of people aged 15 years and older with one of four cancer diagnoses: lung, prostate, breast and colorectal. The exposure group included people with a history of mental health/addiction service contact within the five years before cancer diagnosis, with a subgroup of people with a diagnosis of bipolar disorder, schizophrenia or psychotic disorders. Marginal standardised rates were used to compare emergency presentations (hospital admission within 30 days of cancer diagnosis) in the exposure and comparison groups, adjusted for age, gender (for lung and colorectal cancers), ethnicity, area deprivation and stage at diagnosis.

**Results:**

For all four cancers, the rates of emergency presentation in the fully adjusted models were significantly higher in people with a history of mental health/addiction service use than people without (lung cancer, RR 1.19, 95% CI 1.13, 1.24; prostate cancer RR 1.69, 95% CI 1.44, 1.93; breast cancer RR 1.42, 95% CI 1.14, 1.69; colorectal cancer 1.31, 95% CI 1.22, 1.39). Rates were substantially higher in those with a diagnosis of schizophrenia, bipolar disorder or psychotic disorders.

**Conclusions:**

Implementing pathways for earlier detection and diagnosis of cancers in people with MHSUC could reduce the rates of emergency presentation, with improved cancer survival outcomes. All health services, including cancer screening programmes, primary and secondary care, have a responsibility to ensure equitable access to healthcare for people with MHSUC.

**Supplementary Information:**

The online version contains supplementary material available at 10.1186/s12885-024-12292-9.

## Background

People with mental health and substance use conditions (MHSUC) experience worse health outcomes for many health conditions, including cancer [1–3]. Although the incidence of many cancers is similar to that in the broader population, people with MHSUC have higher mortality from cancer [4–6], a pattern that is consistent across different cancer types and mental health diagnoses [4, 7–9], although disparities are most pronounced for people with diagnoses of schizophrenia and bipolar disorder [2, 10].

Unequal access to health care services and reduced quality of care for people with MHSUC are potential drivers of these differences [5]. Specifically, poorer access to health services that could detect cancers early, such as cancer screening and effective primary care, would mean that incident cancers are more advanced at diagnosis; and poorer access to appropriate and timely treatment would increase mortality [11–13]. Diagnostic overshadowing, where clinicians mistakenly attribute symptoms from a physical condition to a psychiatric cause, can also contribute to delayed cancer diagnoses [14], as can stigma and discrimination against people with MHSUC [10].

Studies have shown that people with cancer and MHSUC are less likely to be screened for cancers [15, 16] and have lower rates of surgery and adjuvant therapy [7, 8, 17]. Higher rates of comorbid physical health conditions in people with MHSUC, such as diabetes and cardiovascular disease, may influence treatment plans and independently affect survival [4, 18].

Pathways to diagnosis are critical for understanding disparities in cancer outcomes. ‘Emergency presentation’ is a measure that can be used to evaluate access to and quality of health services that detect early cancer and can be used to monitor differences in access across populations [19]. These presentations are defined as a diagnosis of cancer occurring within 30 days of an acute or emergency hospital admission (e.g. an emergency presentation with a bowel obstruction leading to a subsequent diagnosis of colorectal cancer). People diagnosed with cancer following an emergency presentation have lower survival rates than those with a non-emergency diagnosis (e.g. through screening or outpatient visits), even after adjusting for stage at diagnosis [20]. Although not all emergency presentations are avoidable, they can be reduced by improvements in health service delivery, screening programme participation and patient awareness of cancer symptoms [21]. In a cross-country comparison of emergency presentation for eight different cancers, Aotearoa New Zealand (NZ) had the highest rate of emergency presentation for all cancers (42.5%), and the 12-month mortality rates for emergency presentation were significantly higher than for non-emergency presentations (e.g. 77% mortality for lung cancer with emergency presentation compared with 45% for lung cancer not diagnosed through emergency presentation) [21].

In NZ, differences in cancer survival for people with MHSUC have been documented for colorectal cancer and breast cancer, with both stage at diagnosis and comorbidities contributing to survival differences [4]. However, the pre-diagnosis pathways leading to these outcomes have not been investigated for people with MHSUC. If differences in emergency presentations were found, this would support the hypothesis that some of the cancer survival disparity between people with and without MHSUC is potentially preventable.

This study used population-level datasets (national mental health service use datasets linked to the national cancer registry and hospital admissions data) to compare rates of emergency presentations of cancer in people with and without a recent history of mental health or addiction service use for four cancer types: lung, prostate, breast and colorectal. We also explored whether emergency presentation rates were higher for people with diagnoses of schizophrenia, bipolar disorder or psychotic disorders.

## Methods

### Study population

We used de-identified national-level linked health datasets to identify the study population of all adults in NZ with cancer alongside exposure status (people with MHSUC). Datasets were linked through an encrypted version of the National Health Index (NHI), a unique identifier assigned to every health service user. The study population included people aged 15 years and older who were included in the New Zealand Cancer Registry over 12 years from 1st July 2006 to 30th June 2018 with lung, breast, colorectal or prostate cancer, using International Classification of Diseases 10th revision (ICD-10) diagnosis codes ICD-10 C34x (lung), ICD-10 C50x (breast), ICD-10 C18x C19x C20x (colorectal) and ICD-10 C61x (prostate). The Cancer Registry records all cancer diagnoses (excluding non-melanoma skin cancers) and stage of disease, if known, via mandatory reporting from laboratories and clinicians.

### Exposure

For this study, people with MHSUC were defined as those who had received treatment from specialist mental health and addiction (SMHA) services in the five years prior to cancer diagnosis. This period of time was chosen to identify a group with significant recent mental health conditions at the time of diagnosis. This group was identified from the Mental Health Information National Collection (MHINC) and the Project for Integration of Mental Health Data (PRIMHD), national data collections on all publicly funded specialist mental health services, both inpatient and outpatient, but excluding primary care mental health contacts. People were included in the exposure group if they had three or more face-to-face activities recorded in PRIMHD/MHINC five years before the incident cancer diagnosis. Face-to-face activities focused on direct person engagement and thus excluded family contacts and support, care co-ordination contacts and non-attended appointments (activity codes included in Appendix 1). Telephone, texts, social media/e-therapy and written correspondence were also excluded. All of those in the exposure group would be classified as having a MHSUC with significant impact on their functioning, as they were seen by public specialist mental health and addiction services. People with organic disorders, intellectual disabilities and developmental disorders, who had no other mental health diagnosis recorded, were excluded (codes for excluded conditions are given in Appendix 1).

A separate grouping of people with diagnoses of bipolar disorder or schizophrenia and related psychotic disorders was created, referred to as ‘schizophrenia/bipolar’. These were people identified as having a ‘principal’, ‘other relevant’, or ‘provisional’ diagnosis of one of these disorders in PRIMHD/MHINC or in the National Minimum Dataset (NMDS), which collects inpatient hospital discharge information, from all public and some private hospitals. Diagnostic codes for bipolar disorder were ICD-10 codes F31x and DSM-IV codes 296.0x, 296.4x, 296.5x, 296.6x, 296.7x, 296.8x,)(OECD, 2018); for schizophrenia and related disorders, ICD-10 codes F20-29, F531 and DSM-IV codes 295x, 297x, 298x, 30,122 (Ministry of Health, 2012).

In the methods and results, we refer to people who use SMHA services, as our analytically defined exposure group, representing a proportion of people with more severe MHSUC. We use MHSUC more generally to refer to all people with these conditions, not all of whom will be included in our analysis, but who are our population of interest.

### Outcome

The emergency presentation outcome was derived from the National Minimum Dataset (NMDS), which records all publicly-funded inpatient admissions in NZ. Emergency presentation was defined as an emergency hospital admission 30 days before a cancer diagnosis, irrespective of the reason or coded diagnosis given for the admission, identified as records coded with an ‘admission type’ of ‘acute admission’ (in contract to waitlist or elective admissions). A 30-day period has been used in previous research as allowing sufficient time for a histological cancer diagnosis and is consistent with other healthcare monitoring measures [21]. 

### Other variables

Age at cancer diagnosis was calculated using date of birth and date of diagnosis from the Cancer Registry. Gender (female and male) and ethnicity (Māori and non-Māori) were taken from the NHI details table. For this analysis, those who reported Māori ethnicity were compared with all other ethnic groups (non-Māori, which included those with missing ethnicity information, *n* = 1782, 1.3% of total). Small numbers of cancers precluded analysis by other ethnic groups.

Deprivation level of the place of residence at time of cancer diagnosis was measured using the NZDep index, a small-area measure derived from 2006 Census data which categorises small areas into deprivation quintiles (1 = least deprived, 5 = most deprived) [22]. 

Stage of cancer disease was based on the Surveillance, Epidemiology and End Results (SEER) Summary Stage method reported in the Cancer Registry and categorised into localised (Stage B), regionally spread (Stage C and D), distant metastases (E), and unknown/unstaged (F).

### Analysis

Data preparation steps were completed in SAS 9.4 (SAS Institute, Cary, NC); statistical analyses were conducted in R 4.2 (R Institute, Vienna, Austria) with marginal standardisation conducted using the marginal effects package.

Analyses were conducted separately for each cancer type. Raw frequencies are presented for number of cancers and number of individuals with an emergency presentation in the prior 30 days, stratified by SMHA service use status. Sociodemographic characteristics are described using frequencies and percentages.

Emergency presentations are summarised initially as percentage of people diagnosed with cancer who met the emergency presentation criteria (within cancer type and SMHA group). These are presented as crude percentages, with no adjustment, and as marginally standardised percentages when adjusted for sociodemographic characteristics.

Marginally standardised rates were calculated to compare emergency presentations between those with and without a history of prior use of mental health or addiction services. This starts as a logistic regression model for the outcome (emergency presentation) modelled according to the main exposure (SMHA status) adjusted for the relevant covariates (age group, gender, ethnicity, stage at diagnosis, NZDep) [23, 24]. A small proportion of individuals were missing NZDep (< 0.5% of records for any given cancer: see results for full summary) and were excluded from these fully-adjusted models.

The model results are then used to marginally standardise the results to a reference population with a covariate profile based on the SMHA group: this represents what the risk of emergency presentation would look like in people not using SMHA services if they had the same characteristics as people with SMHA service use (on the covariates included in the regression model). More formally, the results from the logistic regression model are used to estimate the prevalence of the outcome under two counterfactual scenarios for everyone in the reference population (i.e. holding the confounder/covariate profile constant): one scenario where everyone is treated as having the exposure, and another where no one is considered to have the scenario.

Marginally standardised results are presented as standardised percentages with 95% confidence intervals (95% CI), and as marginally standardised risk ratios (RR) with 95% confidence intervals (calculated as the ratio of emergency presentation risk for the SMHA group divided by the risk in the no-SMHA group).

## Results

Table 1 compares the sociodemographic characteristics of people with and without a history of SMHA service contact across the four cancer types. People who had recently used SMHA services made up between 2% (prostate cancer) to 5% (lung cancer) of those diagnosed with cancer. For all four cancer types, people using SMHA services were younger, more likely to live in deprived areas and more likely to be Māori.

Table 2 details the characteristics of those with schizophrenia/bipolar disorder. The numbers of cancer registrations per year for people with and without MHSUC are provided in Supplementary Table 1 in Additional Files.

**Table 1:** Sociodemographic characteristics of people with lung, prostate, breast and colorectal cancer from 2006–2018, for those with and without specialist mental health or addiction (SMHA) service use in the five years before cancer diagnosis

Characteristics	People not using SMHA services	People using SMHA services	People not using SMHA services	People using SMHA services	People not using SMHA services	People using SMHA services	People not using SMHA services	People using SMHA services
	Lung cancer		Prostate cancer		Breast cancer		Colorectal cancer	
	*n* (%)	*n* (%)	*n* (%)	*n* (%)	*n* (%)	*n* (%)	*n* (%)	*n* (%)
**Total**	22,958 (100.0)	1,125 (100.0)	37,323 (100.0)	794 (100.0)	34,404 (100.0)	1,442 (100.0)	33,615 (100.0)	1,027 (100.0)
**Gender**								
Female	11,006 (47.9)	553 (49.2)	0	0	34,404 (100.0)	1,442 (100.0)	15,977 (47.5)	530 (51.6)
Male	11,952 (52.1)	572 (50.8)	37,323 (100.0)	794 (100.0)	0	0	17,638 (52.5)	497 (48.4)
**Age (years)**								
15–44	314 (1.4)	43 (3.8)	92 (0.2)	4 (0.5)	3,756 (10.9)	229 (15.9)	1,311 (3.9)	108 (10.5)
45–54	1,563 (6.8)	203 (18.0)	2,056 (5.5)	82 (10.3)	8,830 (25.7)	463 (32.1)	2,477 (7.4)	130 (12.7)
55–64	4,564 (19.9)	333 (29.6)	10,473 (28.1)	279 (35.1)	8,650 (25.1)	375 (26.0)	5,584 (16.6)	216 (21.0)
65–74	7,582 (33.0)	322 (28.6)	15,728 (42.1)	272 (34.3)	6,892 (20.0)	180 (12.5)	9,800 (29.2)	231 (22.5)
75+	8,935 (38.9)	224 (19.9)	8,974 (24.0)	157 (19.8)	6,276 (18.2)	195 (13.5)	14,443 (43.0)	342 (33.3)
**Ethnicity**								
Māori	4,056 (17.7)	293 (26.0)	2,310 (6.2)	87 (11.0)	4,124 (12.0)	284 (19.7)	1,731 (5.1)	112 (10.9)
Non-Māori	18,902 (82.3)	832 (74.0)	35,013 (93.8)	707 (89.0)	30,280 (88.0)	1,158 (80.3)	31,884 (94.9)	915 (89.1)
**Deprivation quintile**								
1 (low)	2,803 (12.3)	91 (8.1)	7,794 (20.9)	115 (14.5)	6,791 (19.8)	175 (12.2)	6,013 (17.9)	145 (14.1)
2	3,347 (14.6)	122 (10.9)	7,337 (19.7)	122 (15.4)	6,550 (19.1)	221 (15.4)	6,215 (18.5)	142 (13.8)
3	4,432 (19.4)	202 (18.0)	7,827 (21.0)	138 (17.4)	6,902 (20.1)	310 (21.6)	7,224 (21.6)	211 (20.5)
4	5,690 (24.9)	333 (29.6)	8,129 (21.8)	213 (26.8)	7,308 (21.3)	368 (25.6)	7,919 (23.6)	273 (26.6)
5 (high)	6,601 (28.9)	376 (33.5)	6,177 (16.6)	206 (25.9)	6,776 (19.7)	364 (25.3)	6,137 (18.3)	256 (24.9)
**Stage at diagnosis**								
Localised	1,424 (6.2)	63 (5.6)	84 (10.6)	5,297 (14.2)	18,069 (52.5)	722 (50.1)	7,615 (22.7)	214 (20.8)
Regionally spread	2,954 (12.9)	170 (15.1)	55 (6.9)	3,300 (8.8)	11,336 (32.9)	470 (32.6)	12,769 (38.0)	343 (33.4)
Distant metastases	10,629 (46.3)	493 (43.8)	82 (10.3)	2,158 (5.8)	1,244 (3.6)	49 (3.4)	6,721 (20.0)	258 (25.1)
Unknown	7,951 (34.6)	399 (35.5)	573 (72.2)	26,568 (71.2)	3,755 (10.9)	201 (13.9)	6,510 (19.4)	212 (20.6)

**Table 2:** Sociodemographic characteristics of people with bipolar disorder, schizophrenia or related psychotic disorder and lung, prostate, breast and colorectal cancer from 2006–2018

Characteristics	Lung cancer	Prostate cancer	Breast cancer	Colorectal cancer
	*n* (%)	*n* (%)	*n* (%)	*n* (%)
**Total**	298 (100.0)	135 (100.0)	395 (100.0)	243 (100.0)
**Gender**				
Female	153 (51.3)	0	395 (100.0)	134 (55.1)
Male	145 (48.7)	135 (100.0)	0	109 (44.9)
**Age**				
15–44	10 (3.4)	0 (0.0)	49 (12.4)	16 (6.6)
45–54	47 (15.8)	18 (13.3)	123 (31.1)	31 (12.8)
55–64	102 (34.2)	52 (38.5)	127 (32.2)	66 (27.2)
65–74	100 (33.6)	47 (34.8)	60 (15.2)	69 (28.4)
75+	39 (13.1)	18 (13.3)	36 (9.1)	61 (25.1)
**Ethnicity**				
Māori	74 (24.8)	14 (10.4)	81 (20.5)	23 (9.5)
Non-Māori	224 (75.2)	121 (89.6)	314 (79.5)	220 (90.5)
**Deprivation quintile**				
1 (low)	24 (8.1)	16 (11.9)	43 (10.9)	32 (13.2)
2	35 (11.7)	23 (17.0)	38 (9.7)	23 (9.5)
3	40 (13.4)	25 (18.5)	88 (22.4)	50 (20.6)
4	92 (30.9)	39 (28.9)	114 (29.0)	68 (28.0)
5 (high)	107 (35.9)	32 (23.7)	110 (28.0)	70 (28.8)
**Stage at diagnosis**				
Localised	13 (4.4)	14 (10.4)	200 (50.6)	39 (16.0)
Regionally spread	34 (11.4)	9 (6.7)	131 (33.2)	77 (31.7)
Distant metastases	137 (46.0)	16 (11.9)	18 (4.6)	75 (30.9)
Unknown	114 (38.3)	96 (71.1)	46 (11.6)	52 (21.4)

Table 3 compares emergency presentation in those with and without a history of SMHA service use, and separately for those with schizophrenia/bipolar disorder compared to no history of SMHA service use. Emergency presentation was highest for lung cancer (accounting for over half of all lung cancer diagnoses) and lowest for breast cancer (< 10% of breast cancer diagnoses). For all four cancer types, the proportion of people with emergency presentation was higher in people with SMHA service use than people not using SMHA services. In marginally standardised rates from the fully adjusted models, the rate of emergency presentation was significantly raised for all cancers, indicating that if people not using SMHA services had the same characteristics as people using SMHA services, then rates of emergency presentation would increase 19% for lung cancer (an RR of 1.19 for the relative increase), 69% for prostate cancer, 42% for breast cancer and 31% for colorectal cancer.

For those with schizophrenia/bipolar disorder, these rates were consistently higher. Except for prostate cancer, the difference between the crude and fully adjusted results was small, suggesting that the net effect of confounding factors was minimal.

**Table 3:** Crude and fully adjusted marginal rate ratios for emergency presentation 30 days prior to cancer diagnosis

	Crude model		Fully adjusted model*	
	Proportion (95% CI)	Rate ratio (95% CI)	Proportion (95% CI)	Rate ratio (95% CI)
	Lung cancer			
**No SMH service use**	51.2 (50.5, 51.8)		50.2 (49.4, 50.9)	
**SMH service use (all)**	59.6 (56.8, 62.5)	1.17 (1.11, 1.22)	59.6 (56.9, 62.3)	1.19 (1.13, 1.24)
**No SMH service use**	51.2 (50.5, 51.8)		50.7 (49.9, 51.4)	
**Severe mental illness**	62.1 (56.6, 67.6)	1.21 (1.10, 1.32)	62.1 (56.9, 67.3)	1.23 (1.12, 1.33)
	**Prostate cancer**			
**No SMH service use**	6.6 (6.4, 6.9)		8.4 (8.1, 8.7)	
**SMH service use (all)**	14.2 (11.8, 16.7)	2.15 (1.77, 2.52)	14.2 (12.3, 16.2)	1.69 (1.44, 1.93)
**No SMH service use**	6.6 (6.4, 6.9)		8.4 (8.0, 8.8)	
**Severe mental illness**	17.0 (10.7, 23.4)	2.57 (1.61, 3.53)	17.0 (11.9, 22.2)	2.03 (1.41, 2.65)
	**Breast cancer**			
**No SMH service use**	4.1 (3.9, 4.3)		4.0 (3.8, 4.2)	
**SMH service use (all)**	5.7 (4.5, 6.9)	1.38 (1.08, 1.68)	5.7 (4.6, 6.8)	1.42 (1.14, 1.69)
**No SMH service use**	4.1 (3.9, 4.3)		4.2 (4.0, 4.4)	
**Severe mental illness**	7.1 (4.6, 9.6)	1.72 (1.10, 2.34)	7.1 (4.9, 9.4)	1.70 (1.16, 2.24)
	**Colorectal cancer**			
**No SMH service use**	33.1 (32.6, 33.6)		35.3 (34.7, 35.8)	
**SMH service use (all)**	46.2 (43.1, 49.2)	1.40 (1.30, 1.49)	46.2 (43.3, 49.0)	1.31 (1.22, 1.39)
**No SMH service use**	33.1 (32.6, 33.6)		35.9 (35.3, 36.5)	
**Severe mental illness**	48.6 (42.3, 54.8)	1.47 (1.28, 1.66)	48.6 (42.6, 54.5)	1.35 (1.18, 1.52)

* Adjusted for age, gender (for lung and colorectal cancers), ethnicity, area deprivation and stage at diagnosis. Crude model includes all individuals; fully adjusted models exclude those missing NZDep (< 0.5% for each cancer: Lung excluded *n* = 106 / 24,083, 0.4%; Prostate missing NZDep = 59 / 38,117 = 0.2%; Breast missing *n* = 81 / 35,846 = 0.2%; Colorectal missing *n* = 107 / 34,642 = 0.3%).Logistic regression results are presented in Table 4. For all cancers, higher area deprivation and having a non-localised cancer stage were significantly associated with an increased chance of emergency presentation, as was being Māori for all cancers except prostate. Older age (compared to those aged 55–64 years) also increased the chance of emergency presentation. These patterns were the same for the whole SMHA service use group and the severe mental illness subgroup

**Table 4:** Odds ratios for emergency presentation 30 days prior to cancer diagnosis with all adjustment covariates

Covariate	Lung cancer	Prostate cancer	Breast cancer	Colorectal cancer
	OR (95% CI)	OR (95% CI)	OR (95% CI)	OR (95% CI)
SMHA service use (Ref: none)				
SHMA service use	1.54 (1.35, 1.76)	2.44 (1.89, 3.12)	1.57 (1.21, 2.02)	1.66 (1.45, 1.89)
**Age (Ref: 55–64 years)**				
15–44	1.37 (1.08, 1.75)	3.77 (1.64, 7.69)	0.92 (0.73, 1.16)	2.26 (1.99, 2.56)
45–54	1.13 (1.01, 1.27)	0.91 (0.66, 1.23)	0.75 (0.61, 0.92)	1.08 (0.97, 1.20)
65–74	1.04 (0.96, 1.12)	1.24 (1.07, 1.44)	1.38 (1.13, 1.67)	1.10 (1.02, 1.18)
75+	1.43 (1.33, 1.55)	3.76 (3.26, 4.34)	2.59 (2.18, 3.09)	1.69 (1.58, 1.82)
**Gender (Ref: Female)**				
Male	1.07 (1.01, 1.12)	-	-	0.88 (0.84, 0.92)
**Ethnicity (Ref: Māori)**				
Non-Māori	0.81 (0.75, 0.87)	0.91 (0.76, 1.10)	0.77 (0.65, 0.92)	0.73 (0.65, 0.81)
**Deprivation (Ref: 1 = low)**				
2	1.11 (1.00, 1.23)	1.26 (1.07, 1.48)	1.18 (0.96, 1.46)	1.15 (1.07, 1.25)
3	1.20 (1.09, 1.33)	1.22 (1.04, 1.43)	1.14 (0.93, 1.41)	1.17 (1.08, 1.26)
4	1.19 (1.08, 1.31)	1.24 (1.06, 1.45)	1.26 (1.04, 1.53)	1.22 (1.13, 1.32)
5 (high)	1.37 (1.24, 1.50)	1.61 (1.38, 1.90)	1.60 (1.31, 1.94)	1.43 (1.32, 1.55)
**Stage (Ref: Localised)**				
Regional spread	3.55 (3.00, 4.20)	1.82 (1.34, 2.50)	2.26 (1.89, 2.70)	2.25 (2.10, 2.40)
Distant metastases	13.40 (11.49, 15.70)	46.44 (36.24, 60.36)	70.11 (58.83, 83.83)	5.54 (5.14, 5.97)
Unknown	4.27 (3.66, 5.01)	1.99 (1.57, 2.56)	6.23 (5.20, 7.47)	1.48 (1.37, 1.60)

Odds ratios fully adjusted for all covariates. Fully adjusted models exclude those missing NZDep (< 0.5% for each cancer: Lung excluded *n* = 106 / 24,083, 0.4%; Prostate missing NZDep = 59 / 38,117 = 0.2%; Breast missing *n* = 81 / 35,846 = 0.2%; Colorectal missing *n* = 107 / 34,642 = 0.3%)

## Discussion

This study showed that people using SMHA services have higher rates of emergency presentation for cancer diagnosis, even after accounting for major sociodemographic characteristics compared to those without recent service use. These diagnostic pathways and other factors that contribute to emergency presentation will also contribute to poorer cancer survival rates for people with MHSUC.

People whose cancers are diagnosed after an emergency presentation have worse survival than those who are diagnosed through screening or in non-acute settings [20, 21]. Previous research has found that cancer diagnosed through emergency presentation is associated with type of cancer, stage at diagnosis, deprivation, ethnicity, co-morbidities and older age [21, 25, 26]. This research has indicated that MHSUC status is also related to emergency presentations around cancer diagnosis.

If cancer screening and access to preventive and primary care were the same for all population groups, we would expect similar proportions of people with and without MHSUC being diagnosed with cancer through emergency presentation. Higher rates of emergency presentation in people with MHSUC indicate that these cancers are not being detected in primary care or community settings. While there were some differences in cancer stage at diagnosis at the crude level, even after taking these into account, significant differences in emergency presentation between those with and without MHSUC remained.

### Potentially avoidable factors influencing emergency presentation

Zhou et al. [20] identify potentially modifable factors that affect the risk of emergency presentation for cancer. Factors related to the patient include knowledge and perception of symptoms that might be due to cancer and psychosocial factors influencing help-seeking. These affect both screening uptake and decisions to consult with primary care. Interventions to raise public awareness of potential cancer symptoms and screening programmes and to reduce fear of cancer could improve early detection of cancer and reduce emergency presentations.

However, the effective delivery of information about cancer symptoms and services will be insufficient for people with MHSUC who are reluctant to seek help because of past experiences of discrimination [27, 28]. Bias against people with MHSUC from healthcare professionals is well documented and contributes to poorer physical health outcomes through mechanisms spanning from reduced help-seeking to reduced provision of timely and appropriate investigation of symptoms and treatment of disease [27–32]. In NZ, people with MHSUC who sought healthcare for physical symptoms commonly reported having their symptoms dismissed or ignored and that diagnosis and management of physical conditions was delayed [33]. This is known as diagnostic overshadowing, where physical symptoms are misattributed by health professionals to mental health conditions, and it occurs in all healthcare settings [34].

Other potentially avoidable factors for emergency presentation related to the healthcare system include affordability, availability and quality of primary care services [20]. In NZ, ethnic differences in rates of emergency presentation for lung cancer have been found, with Māori and Pacific peoples with lung cancer having higher rates of emergency presentation than people of European ethnicity, even after adjusting for stage at diagnosis [19]. The differential experience of barriers to primary healthcare is one important explanation for this. In the 2021/22 New Zealand Health Survey (NZ’s major national health survey), Māori reported more than two-fold higher rates compared to non-Māori of not visiting a GP in the last 12 months because of owing money (RR = 2.78), not having transport to get there (RR = 2.45) or fear or dislike of the GP (RR = 2.88) [35]. The New Zealand Health Survey has not examined barriers to primary healthcare for people with MHSUC, but differences in quality of primary healthcare for people with a current mental health condition have been reported from other NZ surveys [36]. People with MHSUC are also more likely to experience cost as a barrier to primary healthcare, because of the known association between MHSUC and socioeconomic disadvantage [37]. Although primary care services are largely publicly funded, most service providers charge copayments for visits, and prescriptions have historically been additional out-of-pocket expenses. However, emergency departments are publicly funded with no co-payment and may be more accessible for those who are unable to afford or access primary healthcare. Although we adjusted for area deprivation in the analysis, residual confounding by socioeconomic status could explain some of the difference in emergency presentations between those with and without MHSUC.

Research from the UK on people diagnosed with cancer as an emergency has found that the majority had at least one primary care consultation in the 12 months prior to diagnosis about symptoms relevant to the cancer [38] and as many as one fifth of those with an emergency diagnosis of colorectal cancer had at least one red-flag symptom [39], suggesting at least some of the emergency presentations could have been prevented. Furthermore, general practices in the UK with higher scores from a quality performance framework had lower rates of emergency presentation for cancer [40]. In NZ, emergency diagnosis of lung cancer was lower in those who had had a primary care consultation in the three months prior to diagnosis, a phenomenon observed across ethnic groups [19]. More research is needed into the patterns of primary care consultation in people with MHSUC with cancer diagnosed after emergency presentation.

Timeliness of diagnostic services, including reporting on and follow-up of test results, is another avoidable risk factor for emergency presentation [20]. General practitioners (GPs) in NZ have much lower and slower access to diagnostic tests and specialist advice than in other jurisdictions [41, 42]. While this helps explain why rates of emergency presentation for cancer are high in NZ overall, it may or may not relate to why rates are even higher in people with MHSUC.

### Strengths and limitations

This was a population-based study that used national datasets with comprehensive information on cancer and hospitalisation outcomes. Using the databases that record receipt of treatment from specialist mental health and addiction services allowed the identification of people with more severe MHSUC, but also means that the results are not necessarily applicable to those with less severe conditions, who are treated in the community. Although the majority of people with serious MHSUC conditions access specialist services in the past 12 months [43] this analysis will miss a proportion of those for whom MHSUC has had a significant and recent impact on their lives. The potential misclassification of these people with MHSUC who have not engaged with specialist services may have reduced the relative difference between those with and without MHSUC, as defined in this study.

The definition of emergency presentation for cancer used in this and in other studies does not attempt to establish that the emergency presentation 30 days before the cancer diagnosis was unquestionably caused by the cancer. There may be some people who attended ED for a reason unrelated to the subsequent cancer diagnosis. However, at a population level, this definition has effectively worked to identify large differences in outcomes between those with and without emergency presentation [20]. Due to known inaccuracies in hospital diagnostic coding, this approach is deemed to be more robust than trying to define a cancer-specific spectrum of emergency presentation codes [21].

Differential participation in screening programmes by people with and without MHSUC could contribute to the difference in emergency presentation, although a national organised screening programme was only in place for breast cancer during the time of this study. There is no programme for lung cancer screening, screening for prostate cancer is opportunistic and colorectal cancer screening was only fully implemented nationwide in 2022 [44]. Future research into the accessibility and timeliness of breast and colorectal cancer screening in people with MHUSC is recommended.

Comorbidity is a risk factor for emergency presentation that is associated with poorer cancer survival [4, 26]. This study did not adjust for the presence of comorbidities, but this is likely to be a factor in delayed cancer diagnosis in people with MHSUC who experience diagnostic overshadowing.

In NZ, the proportion of cancer diagnosed through emergency presentation ranges from 19.8% for rectal cancer to 60.4% for pancreatic cancer [21]. We examined differences in emergency presentation among those with and without MHSUC only for the most commonly registered cancers, excluding other cancer groups due to relatively small numbers. Other NZ research has documented poorer cancer survival and higher mortality in Māori, as well as higher rates of emergency presentation [19, 45, 46]. Given the double jeopardy of ethnicity and mental health for many physical health outcomes [6, 47], further research into emergency presentation for cancer in Māori with MHSUC is warranted.

The high proportion of cancers with unknown stage, especially for prostate cancer, means that differences in stage between populations are difficult to interpret. The emergency presentation is a preferable indicator for monitoring of cancer outcomes when information on cancer stage is incomplete.

### Implications

The results of this study strongly suggest that the health system has a role in contributing to unequal cancer survival for people with MHUSC and that at least a proportion of emergency presentation for cancer in people with MHSUC is avoidable.

The potential for screening programmes to reduce emergency presentations has been demonstrated for colorectal cancer [25, 48]. Currently in NZ, population-based screening for breast and colorectal cancer is free for eligible age groups, but programme participation rates for people with MHSUC are unknown. However, from the international literature, the prevalence of most types of cancer screening is significantly reduced in people with MHSUC [16]. People with MHSUC have been identified as a priority population in New Zealand’s Cancer Action Plan for achieving equity in outcomes, including access to screening [49]. Monitoring of access to screening for people with MHSUC and codesign of screening programmes is required to ensure that screening acts to decrease inequities in cancer diagnosis and survival. We should also monitor and minimise differential harms from screening, particularly potential exacerbation of mood disorders from false positive screening results in people with MHSUC.

Improved access to primary care is another factor that could reduce emergency presentation for those with MHSUC but more research is needed to establish the most important barriers to early diagnosis and the contribution of diagnostic overshadowing. A trusted relationship with a GP is a major enabler of early lung cancer diagnosis for Māori in NZ [42] and also an enabler of primary healthcare access for people with MHSUC in general [50, 51]. Both physical and mental healthcare professionals, in primary and secondary care, need to be aware that people with MHSUC are at higher risk of delayed cancer diagnosis. To mitigate this, all healthcare professionals need to promote and facilitate screening in this population and support people with MHSUC and their family/whānau to recognise and seek help for symptoms that may indicate cancer.

In England, reductions in emergency presentation for cancer have been at least partially attributed to health system changes that include provision and uptake by GPs of an urgent referral pathway for suspected cancer [52] alongside publication of guidelines from the National Institute for Health and Care Excellence on referral of suspected cancer in primary care [53]. Other health system changes with the potential to reduce excess mortality in people with MHSUC include improved care coordination and integration of mental and physical healthcare, interventions to reduce discrimination and diagnostic overshadowing by health professionals and services to support the reduction of risk factors (e.g. tobacco cessation programmes) [10, 14].

NZ’s Cancer Control Agency (Te Aho o Te Kahu) currently monitors emergency presentations for several cancers (colorectal, prostate, pancreatic and lung) by region, age, gender, ethnicity and deprivation [54]. Adding MHSUC status (or use of SMHA services) to this monitoring framework would allow assessment of the impact of interventions for improving cancer diagnosis and treatment in people with MHSUC.

## Conclusions

Using national-level data, we found that people with MHSUC had higher rates of cancer diagnosed after emergency presentation than those without, even after adjusting for confounding factors. Emergency presentation is a contributing factor to poorer cancer survival in people with MHSUC. The healthcare system has a vital role in addressing health inequities and improving access to timely and non-discriminatory care for priority groups. This research demonstrates that early detection and diagnosis of cancer in people with MHSUC is needed to improve cancer survival rates. This will require multifaceted improvements in cancer screening, primary care and diagnostic services, referral pathways and outcome monitoring for people with MHSUC across the cancer continuum.

### Electronic supplementary material

Below is the link to the electronic supplementary material.

Supplementary Material 1

## Data Availability

The primary data that support the findings of this study can be requested from Te Whatu Ora/ Health New Zealand National Collections team, subject to their data access policies. The datasets used and/or analysed during the current study are available from the corresponding author on reasonable request.

## Abbreviations

CI: Confidence interval
GP: General practitioner
ICD: International Classification of Diseases
MHSUC: mental health or substance use condition
MHINC: Mental Health Information National Collection, a national collection of mental health and addiction services data from July 2000 to June 2008, replaced by PRIMHD
National Health Index (NHI): a unique identifier assigned to every health service user
NMDS: National Minimum Dataset, a national collection of hospitalisation data
NZ: Aotearoa New Zealand
NZ Dep: New Zealand Deprivation Index, a measure of area deprivation
PRIMHD: Project for Integration of Mental Health Data, a national collection of mental health and addiction services data, which replaced MHINC from July 2008
RR: Relative risk
SEER Summary Stage: Cancer staging classification used by the Surveillance, Epidemiology and End Results (SEER) Program at the National Cancer Institute, National Institute of Health (United States)
SMHA: specialist mental health and addiction (services)
