# Supplementary material for: Cancer diagnosis after emergency presentations in people with mental health and substance use conditions: a national cohort study
Source: BMC Cancer. 2024 Apr 30;24:546. doi: 10.1186/s12885-024-12292-9 (PMC11062004; doi:10.1186/s12885-024-12292-9)
Supplement: Supplementary file 1 — Supplementary Material 1 [file 12885_2024_12292_MOESM1_ESM.docx]

# Supplementary Table

Supplementary Table 1 Cancer registrations by year

| **Registration year** | **All people not using SMHA services** | **All people using SMHA services** | **People with severe mental illness** |
| --- | --- | --- | --- |
|  | **n (%)*** | **n (%)*** | **n (%)*** |
|  | **Lung cancer** | | |
| **2006/07** | 1,598 (7.0) | 60 (5.3) | 14 (4.7) |
| **2007/08** | 1,723 (7.5) | 57 (5.1) | 16 (5.4) |
| **2008/09** | 1,799 (7.8) | 73 (6.5) | 13 (4.4) |
| **2009/10** | 1,886 (8.2) | 77 (6.8) | 15 (5.0) |
| **2010/11** | 1,910 (8.3) | 75 (6.7) | 22 (7.4) |
| **2011/12** | 1,854 (8.1) | 95 (8.4) | 24 (8.1) |
| **2012/13** | 1,930 (8.4) | 90 (8.0) | 29 (9.7) |
| **2013/14** | 1,983 (8.6) | 116 (10.3) | 21 (7.0) |
| **2014/15** | 2,052 (8.9) | 136 (12.1) | 42 (14.1) |
| **2015/16** | 2,057 (9.0) | 125 (11.1) | 32 (10.7) |
| **2016/17** | 2,098 (9.1) | 128 (11.4) | 38 (12.8) |
| **2017/18** | 2,068 (9.0) | 93 (8.3) | 32 (10.7) |
|  | **Prostate cancer** | | |
| **2006/07** | 2,730 (7.3) | 39 (4.9) | 8 (5.9) |
| **2007/08** | 2,850 (7.6) | 61 (7.7) | 15 (11.1) |
| **2008/09** | 3,163 (8.5) | 44 (5.5) | 8 (5.9) |
| **2009/10** | 3,115 (8.3) | 57 (7.2) | 9 (6.7) |
| **2010/11** | 2,879 (7.7) | 65 (8.2) | 10 (7.4) |
| **2011/12** | 3,084 (8.3) | 66 (8.3) | 15 (11.1) |
| **2012/13** | 2,959 (7.9) | 73 (9.2) | 13 (9.6) |
| **2013/14** | 3,061 (8.2) | 73 (9.2) | 11 (8.1) |
| **2014/15** | 3,025 (8.1) | 67 (8.4) | 9 (6.7) |
| **2015/16** | 3,074 (8.2) | 78 (9.8) | 9 (6.7) |
| **2016/17** | 3,421 (9.2) | 86 (10.8) | 16 (11.9) |
| **2017/18** | 3,962 (10.6) | 85 (10.7) | 12 (8.9) |
|  | **Breast cancer** | | |
| **2006/07** | 2,460 (7.2) | 76 (5.3) | 20 (5.1) |
| **2007/08** | 2,534 (7.4) | 94 (6.5) | 24 (6.1) |
| **2008/09** | 2,650 (7.7) | 103 (7.1) | 44 (11.1) |
| **2009/10** | 2,710 (7.9) | 114 (7.9) | 29 (7.3) |
| **2010/11** | 2,668 (7.8) | 114 (7.9) | 24 (6.1) |
| **2011/12** | 2,822 (8.2) | 109 (7.6) | 34 (8.6) |
| **2012/13** | 2,894 (8.4) | 116 (8.0) | 27 (6.8) |
| **2013/14** | 2,980 (8.7) | 126 (8.7) | 40 (10.1) |
| **2014/15** | 3,093 (9.0) | 161 (11.2) | 58 (14.7) |
| **2015/16** | 3,226 (9.4) | 166 (11.5) | 31 (7.8) |
| **2016/17** | 3,110 (9.0) | 128 (8.9) | 37 (9.4) |
| **2017/18** | 3,257 (9.5) | 135 (9.4) | 27 (6.8) |
|  | **Colorectal cancer** | | |
| **2006/07** | 2,625 (7.8) | 63 (6.1) | 15 (6.2) |
| **2007/08** | 2,641 (7.9) | 89 (8.7) | 20 (8.2) |
| **2008/09** | 2,579 (7.7) | 76 (7.4) | 26 (10.7) |
| **2009/10** | 2,801 (8.3) | 83 (8.1) | 19 (7.8) |
| **2010/11** | 2,744 (8.2) | 63 (6.1) | 12 (4.9) |
| **2011/12** | 2,786 (8.3) | 90 (8.8) | 23 (9.5) |
| **2012/13** | 2,852 (8.5) | 71 (6.9) | 17 (7.0) |
| **2013/14** | 2,891 (8.6) | 107 (10.4) | 22 (9.1) |
| **2014/15** | 2,949 (8.8) | 94 (9.2) | 21 (8.6) |
| **2015/16** | 2,951 (8.8) | 99 (9.6) | 23 (9.5) |
| **2016/17** | 2,912 (8.7) | 93 (9.1) | 27 (11.1) |
| **2017/18** | 2,884 (8.6) | 99 (9.6) | 18 (7.4) |

*Proportion of total cancer type from each year
